# Diagnostic challenges of hypomelanosis of Ito: Subtle Blaschko-linear hypopigmentation in Fitzpatrick type I

**DOI:** 10.1016/j.jdcr.2025.12.029

**Published:** 2025-12-26

**Authors:** Brooke Swain, Elizabeth Swain, Roberta Swain

**Affiliations:** a Vanderbilt University School of Medicine, Nashville, Tennessee; b Wake Forest University School of Medicine, Winston-Salem, North Carolina; c University of South Alabama Dermatology, Mobile, Alabama

**Keywords:** case report, hypomelanosis of ito, neurocutaneous disorder

A 13-year-old female, Fitzpatrick skin type I, presented for evaluation of linear hypopigmented patches along her right arm, first noticed by her mother at birth. Due to her fair complexion, these lesions were difficult to appreciate and were only visible with flushing or sun exposure. The patches were unchanged in size throughout development, and there was no history of blistering or hyperpigmentation. The patient’s medical history was significant for epilepsy, characterized by absence, myoclonic, and generalized tonic-clonic seizures, with onset at age 10. Brain MRI revealed no structural abnormalities. Given the combination of recurrent seizures and a rash of unknown significance, the patient was referred for evaluation by a board-certified dermatologist at age 13. On physical examination, hypopigmentation was not visible with ambient lighting. However, under Wood’s lamp examination, hypopigmented linear streaks and patches were noted on the patient's right arm, upper arm, shoulder, and neck (Fig 1). Biopsy was not necessary to confirm the diagnosis. What is the correct diagnosis for this patient?

**A.** Hypomelanosis of Ito (HI)
**B.** Nevus depigmentosus
**C.** Vitiligo
**D.** Lichen sclerosis
**E.** Incontinentia pigmenti

**Fig 1:**
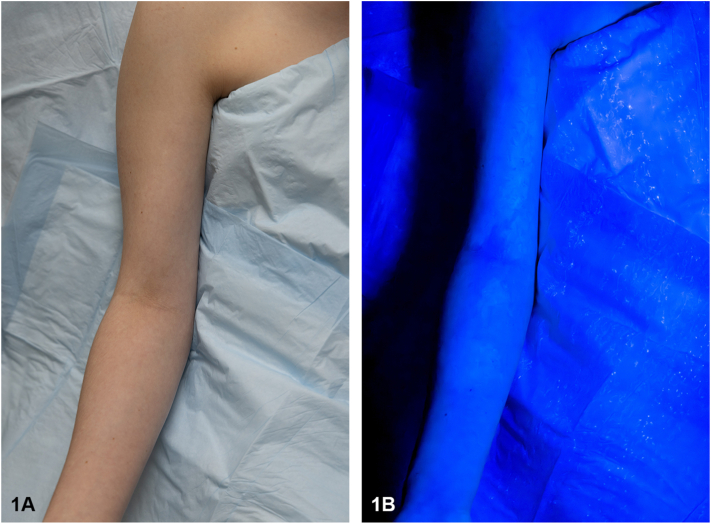
Streaked hypopigmentation along *lines* of Blaschko is only observed under Wood’s lamp fluorescence. **(A)** Clinical appearance under ambient room lighting. **(B)** Corresponding findings visualized under Wood's lamp illumination.

**Answer: A**

Discussion: Hypomelanosis of Ito (HI), also known as incontinentia pigmenti achromians, is a neurocutaneous disorder characterized by hypopigmented whorls or streaks following the lines of Blaschko.^1^ It is associated with variable systemic findings involving the central nervous system, eye, and musculoskeletal system, most commonly intellectual disability or epilepsy.^2^, ^3^, ^4^ However, current literature suggests that most patients with pigmentary mosaicism have low rates of neurologic abnormalities, with only 15% of patients affected.^5^ Skin findings are typically present at birth but become more apparent within the first year of life, especially with sun exposure and tanning.^3^^,^^4^ Reported cases have been diagnosed through visual identification on physical examination or with a Wood’s Lamp.^2^^,^^4^ We present a case of a 13-year-old female, Fitzpatrick skin type I who presented with faint, hypopigmented patches on physical examination and was found to have whorls along Lines of Blashko consistent with HI upon Woods Lamp visualization.

A clinical diagnosis of HI was made based on the patient’s characteristic skin findings, Wood’s lamp examination, and neurological history. Given the patient’s seizure history, the patient was referred to ophthalmology and otolaryngology to evaluate for retinal abnormalities and sensorineural hearing loss. No anomalies were detected. Ongoing care was provided by pediatric neurology for seizure management. This report advocates for early detection of HI in patients with extracutaneous manifestations of HI, particularly when cutaneous evidence is minimal or inconspicuous, underscoring the need for continued vigilance in clinical practice. HI may be underdiagnosed in lighter skin tones, and Wood’s Lamp examination is an effective diagnostic tool when lesions are subtle in ambient lighting.

## Conflicts of interest

None disclosed.
